# Pattern of Independent Factors Influencing Preterm Infants' Chance of Survival to Discharge Without Any Significant Morbidities

**DOI:** 10.7759/cureus.95264

**Published:** 2025-10-23

**Authors:** Anna Petrova, Rajeev Mehta

**Affiliations:** 1 Pediatrics, Rutgers Robert Wood Johnson Medical School, New Brunswick, USA; 2 Neonatology, Rutgers Robert Wood Johnson Medical School, New Brunswick, USA

**Keywords:** bronchopulmonary dysplasia, intraventricular hemorrhage, morbidities, preterm infants, retinopathy of prematurity, survival to discharge

## Abstract

Background

Recognizing the factors that contribute to significant morbidity in preterm infants is essential for effective clinical management and parental counseling. However, current knowledge in this area remains limited.

Methods

This retrospective single-center study aimed to identify the predictors of bronchopulmonary dysplasia (BPD), intraventricular hemorrhage (IVH), and retinopathy of prematurity (ROP) in surviving infants born between 22 and 32 weeks of gestation. Of the 443 infants studied, 269 did not have any of these conditions, while 174 had at least one. Among the latter group, 110 infants had one, and 64 had more than one disease. We conducted group-based comparisons of 33 maternal, intrapartum, and neonatal factors, which helped identify 15 potentially significant predictors. These were included in regression models to identify those that predicted survival without BPD, IVH, and ROP, as well as survival with one or more of these morbidities. The models presented odds ratios (ORs) along with 95% confidence intervals (CIs). A p-value of less than 0.05 was considered statistically significant.

Results

The likelihood of survival without BPD, IVH, and ROP was decreased in infants born with a gestational age of 22-27 weeks (OR 0.68, 95% CI 0.48, 0.97), who were mechanically ventilated (OR 0.48, 95% CI 0.35, 0.98), or diagnosed with thrombocytopenia (OR 0.52, 95% CI 0.35, 0.79), sepsis (OR 0.74, 95% CI 0.56, 0.97), or necrotizing enterocolitis (NEC; OR 0.28, 95% CI 0.22, 0.91), but was increased in the presence of maternal pre-eclampsia (OR 1.44, 95% CI 1.05, 1.97). The presence of intrapartum antibiotics, mechanical ventilation, thrombocytopenia, and NEC predicted the finding of a single morbidity, whereas a gestational age of 22-27 weeks, mechanical ventilation, and thrombocytopenia predicted more than one morbidity in these infants.

Conclusion

Extreme prematurity, mechanical ventilation, and thrombocytopenia are important factors, and their presence or absence can help distinguish infants who will survive without BPD, IVH, and ROP from those who will have two or three morbidities. Further validation of these findings is necessary before their application in clinical practice.

## Introduction

There has been a significant improvement in survival and rate of morbidities in prematurely born infants [1]. However, infants born at extremely preterm gestation continue to remain at an elevated risk of being diagnosed with at least one major morbidity [2], which negatively impacts their quality of life [3] and increases health expenditure [1,4]. One of the challenges associated with delivery at extremely preterm gestation is predicting the development of significant morbidities in the surviving neonate. A few reports [5-7], inconsistent in their gestational age (GA)-based inclusion criteria, selection of predictor factors, and types of the studied morbidities, have focused on identifying factors beyond GA and birth weight (BW) to predict significant health problems in preterm babies. A large study, which included data from Spain (SENI500) and the Latin American Neonatal Networks (NEOCOSUR), reported a reduced risk of developing intraventricular hemorrhage (IVH grade 3-4), periventricular leukomalacia (PVL), bronchopulmonary dysplasia (BPD), retinopathy of prematurity (ROP), necrotizing enterocolitis (NEC), and late-onset sepsis (LOS) in infants born at a GA of 24 to 30 weeks if they were female, had higher Apgar scores, and were not intubated [5]. Simultaneously, the risk increased for infants born from multiple pregnancies, small for gestational age (SGA), and within the Latin American Network [5]. The Canadian Neonatal Network's study of infants born at a GA of 22-32 weeks highlights a higher probability of surviving without BPD, IVH (grade 3-4), PVL, and ROP for females and those born at advanced GA and BW [6]. A study from California utilized the Statewide Health Planning and Development database to construct resiliency scores for estimating the probability of survival without BPD, NEC, IVH (grades 3-4), PVL, and ROP in infants born at less than 32 weeks [7]. The authors stated that resilience scores predict morbidity in preterm infants more accurately than risk assessments. Unfortunately, the lack of an automated score calculator could restrict adherence if the resilience scores are implemented in neonatal practice [8].

We designed this single-center retrospective study to highlight the importance of predicting significant health challenges in the survival of preterm infants. Our primary goal was to identify whether maternal, intrapartum, and neonatal factors independently predispose to the survival of infants born between 22 and 32 weeks of GA without experiencing any BPD, IVH, and ROP. Our second goal was to explore the factors that predict the diagnosis of one or more of these morbidities. Existing evidence suggests that the number of morbidities is a key factor linked to an increased risk of adverse neurodevelopmental outcomes [9]. To enhance clinical management and empower family education, it is essential to understand the factors that lead to the development of serious preterm morbidities.

## Materials and methods

This study utilized a retrospective cohort design that focused on surviving infants with GA less than 33 weeks of completed gestation between January 2007 and January 2016 and admitted to the 37-bed Level III Neonatal Intensive Care Unit (NICU). The study was approved by the Rutgers Robert Wood Johnson Institutional Review Board (ID: 20160000233) on 03/04/2016.

Data characteristics

We used GA as specified by the obstetrician to select neonates from NeoData, a data system designed to assist in the daily patient management of neonates, and a standardized data collection tool to gather the documented maternal, antenatal, and intrapartum variables from the electronic medical records (EMR). Because of the low rates of chronic and maternal infection-related morbidity, we defined categories based on whether at least one morbidity was documented. We categorized maternal age into two groups: 35 years and older or less than 35 years, owing to the heightened risk of adverse fetal conditions associated with advanced maternal age, defined as 35 years and older [10]. We classified BW as extremely low birth weight (ELBW) if the neonate weighed less than 1,000 grams [11] and categorized infants as SGA if their birth weight fell below the 10th percentile for their GA [12]. We defined a low Apgar score as a score of less than three at one minute and less than five at five minutes [13]. Patients with thrombocytopenia were identified if their platelet count was at least once less than 150,000/microL [14]. The diagnosis of sepsis was used if the blood culture or cerebrospinal fluid (CSF) was assessed as positive for bacterial pathogens, and NEC was utilized without considering stage [15].

Definition of morbidities

The primary outcomes were focused on morbidities documented in the EMR: (i) IVH of any grade, including low grade [16], because it is linked with neurodevelopmental impairment in infants born prematurely [17]; (ii) BPD of any grade because of the questionable validity of the existing definitions of this most common preterm birth-related pathology [18,19]; and (iii) ROP, if it was diagnosed and monitored by the NICU ophthalmologist.

Outcome definition

We defined the diagnosis-specific groups of surviving infants as (i) without any IVH, BPD, and ROP (Group 1); (ii) with at least one of these morbidities (Group 2); (iii) with only one of the studied morbidities (Group 2A); and (iv) with more than one morbidity (Group 2B).

Sample size

We calculated the sample size needed to create a binary stepwise logistic regression model to predict survival to discharge without BPD, IVH, and ROP. The formula used was N = 10 * k / p, where N is the total number of infants studied (N=443), 10 is the events per variable (EPV), k is the number of independent variables, and p is the fraction of infants who were discharged without all three studied morbidities (0.614). We estimated that up to 27 independent variables (k) could be included in the model with a sample size of 436 subjects.

Statistical analysis

We identified differences in categorical and continuous variables among neonates in Group 1 vs. Group 2, Group 1 vs. Group 2A, and Group 1 vs. Group 2B using the chi-square test and analysis of variance (ANOVA), and the Mann-Whitney U test for nonparametric variables when necessary. Identified significant factors were incorporated as independent variables in stepwise logistic regression models, using a backward elimination approach for predictors. In Model 1, we focused on the factors predicting survival without any morbidity; in Model 2, we examined survival with one morbidity, and in Model 3, we assessed survival with more than one of the studied morbidities. GA and BW were included in the models as categorical variables (22-27 vs. 28-32 weeks and ELBW vs. >1,000 grams) because the variance inflation factor (VIF) between GA and BW is 1.0. Data are presented as proportions with 95% confidence intervals (95% CI), parametric continuous data as means with 95% CI, and nonparametric data as medians with interquartile ranges (IQR). The outcomes from each model were articulated via odds ratios (OR) with 95% Confidence Intervals (95% CI). Additionally, we employed receiver operating characteristic (ROC) analysis, represented by the area under the curve (AUC), to evaluate the discriminatory power between dependent groups in each model. We used the bootstrap technique to assess the internal validity of the stepwise logistic regression model. The AUC is presented with 95% CI. It was considered acceptable if the AUC was 0.7-0.8, excellent if 0.8-0.9, and outstanding if more than 0.9. P-values at less than 0.05 were considered statistically significant. We used TIBCO Statistica™ 14.01.25, 2020 (TIBCO Software Inc., Palo Alto, California, US). This software facilitates the execution of a wide range of statistical analyses, including regression modeling.

## Results

Of the 498 selected infants, 52 who died and three with incomplete birth data were excluded. Of the 443 surviving neonates, 269 (60.7%, 95% CI 56.0%, 65.31%) were free from any IVH, BPD, and ROP (Group 1), while 174 (39.3%, 95% CI 34.7%, 44.0%) were diagnosed with at least one disease (Group 2), including 110 (63.2%, 95% CI 55.6%, 70.4%) with one (Group 2A) and 64 (36.8%, 95% CI 29.6%, 44.4%) with two (n=50) or all three (n=14) morbidities (Group 2B). IVH was recorded in 70 (45.4%, 95% CI 37.9%, 53.1%), BPD in 115 (66.1%, 95% CI 58.5%, 73.1%), and ROP in 58 (33.3%, 95% CI 26.4%, 40.9%) infants.

**Figure 1:**
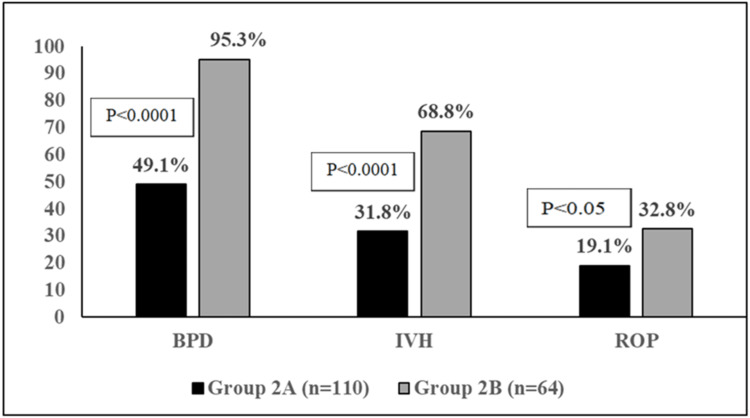
The proportion of BPD, IVH, and ROP cases in Group 2A and Group 2B BPD: Bronchopulmonary dysplasia; IVH: Intraventricular hemorrhage; ROP: Retinopathy of prematurity; Group 2A: with only one of the studied morbidities; Group 2B: with more than one morbidity.

Figure 1 illustrates the larger proportion of BPD cases and the less frequent diagnosis of IVH and ROP in Groups 2A and 2B. 

Disease-specific rates of IVH, BPD, and ROP in the 443 infants studied were 15.8% (95% CI 12.5%, 19.5%), 26.0% (95% CI 22.0%, 30.3%), and 13.1% (95% CI 10.1%, 16.6%), respectively.

GA-based distribution of morbidity and risk factors

**Figure 2:**
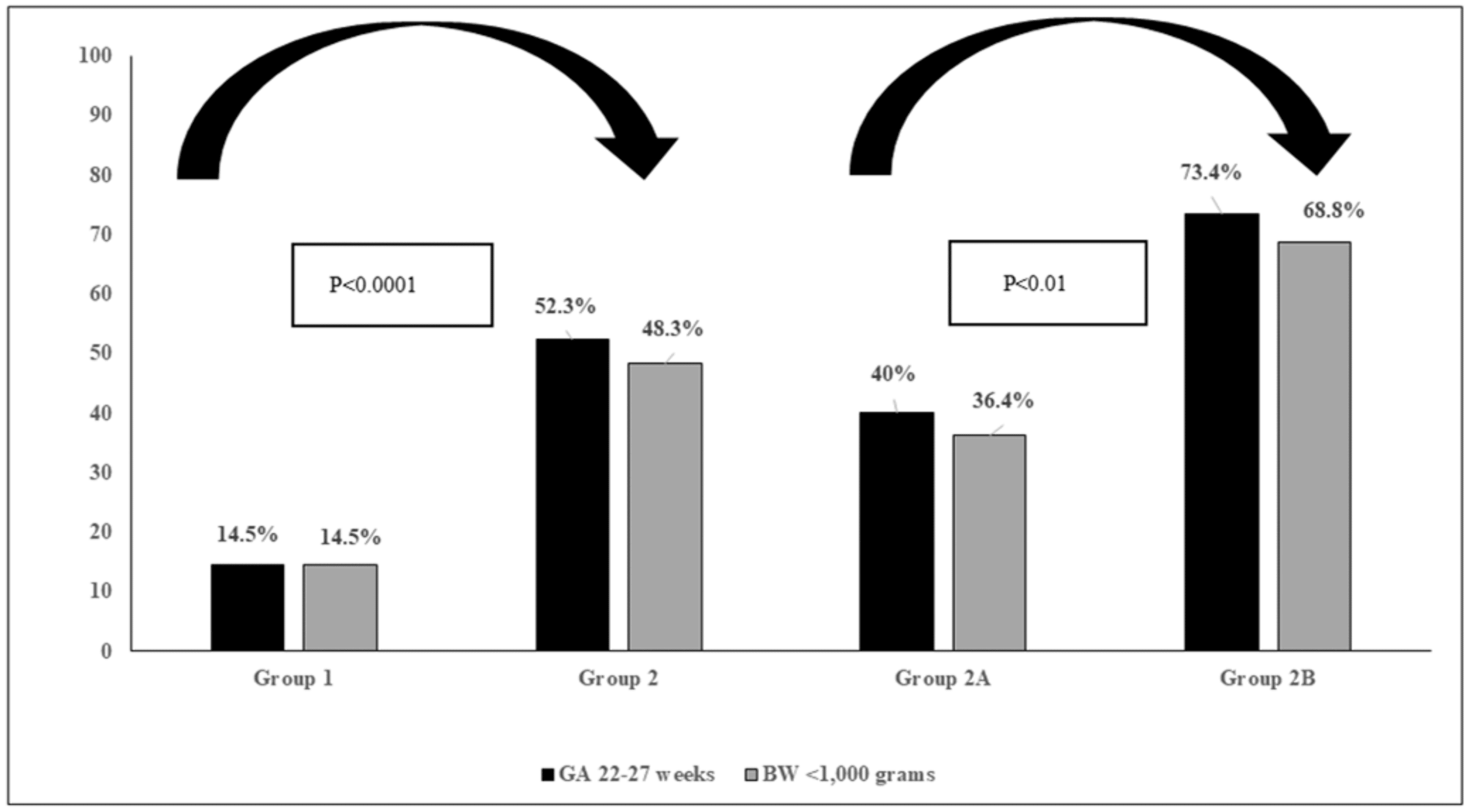
Proportion (%) of infants with gestational age (GA) of 22-27 weeks and birth weight (BW) <1,000 grams in the studied groups

Out of the 443 infants in the study, 29.3% (95% CI 25.2%, 33.8%) were extremely preterm (GA 22-27 weeks), and 27.8% (95% CI 23.6%, 32.2%) were born with ELBW (BW<1,000 grams). Figure 2 demonstrates the group-based difference in the proportion of infants born with GA 22-27 weeks and BW <1,000 grams.

**Figure 3:**
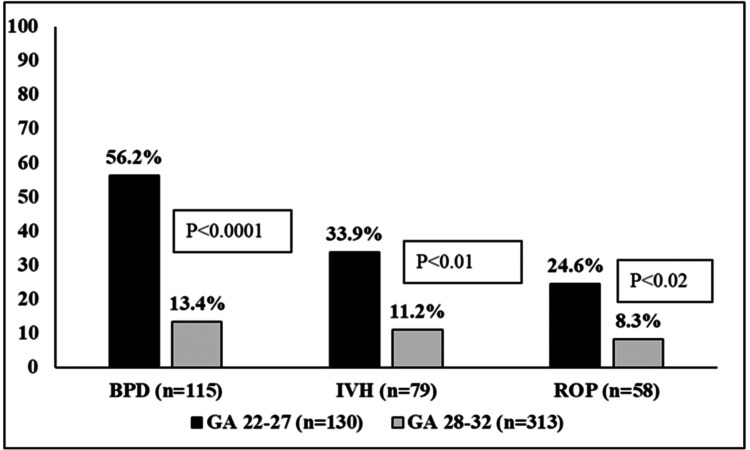
Rates (%) of BPD, IVH, and ROP in infants born with GA of 22-27 and 28-32 weeks GA: Gestational age; BPD: Bronchopulmonary dysplasia; IVH: Intraventricular hemorrhage; ROP: Retinopathy of prematurity

These infants were less likely to have a single disease diagnosed than those with more than one of the studied morbidities (P<0.01). Data in Figure 3 shows that IVH, BPD, and ROP rates were three to four times higher in infants born with a GA of 22-27 weeks as compared to those born with a GA of 28-32 weeks.

Group-based comparison of the studied factors is shown in Table 1.

**Table 1:** Group-based comparison of maternal, antenatal, and neonatal factors GA: Gestational age; SGA: Small for Gestational Age; IUGR: Intrauterine Growth Restriction; ELBW: Extremely Low Birth Weight; Maternal chronic morbidity: defined on EMR (diabetes, hypertension, asthma, hyper/hypothyroidism, seizures, coagulopathy, leukemia, and systemic connective diseases); Maternal infection morbidity: defined on EMR (urinary tract infection (UTI), sexually transmitted infection (STI), group B strep (GBS));  PPROM: Preterm premature rupture of membranes; DART: Dexamethasone: A Randomized Trial; FHR: Fetal heart rate;  MV: Mechanical Ventilation; CPAP: Continuous Positive Airway Pressure; PDA: Patent Ductus Arteriosus; NEC: Necrotizing Enterocolitis; M: Mean +/-Standard Deviation (SD); *P<0.05-0.02; **P<0.01; ***P<0.001.

Factors	Group 1 (N=269)	Group 2 (N=174)	Group 2A (N=110)	Group 2B (N=64)
					GA (M+/-SD, wks)	29.6 +/- 1.9	27.2 +/- 2.4***	27.9 +/- 2.3***	26.0 +/- 2.2***
BW (M+/-SD, g)	1371 +/- 377	1044 +/- 353***	1137 +/- 346***	887 +/- 309***
Male sex	50.4% (135/268)	48.0% (82/171)	46.8% (51/109)	50.0% (31/62)
Maternal age >35 y	22.4% (64/262)	22.2% (38/171)	25.5% (28/110)	16.4% (10/61)
White race	33.2% (84/268)	38.5% (67/174)	44.6% (49/110)	28.2% (18/64)
Medicaid/uninsured	46.6% (124/266)	45.1% (78/173)	40.4% (44/109)	53.1% (34/64)
No prenatal care	4.5% (12/269)	4.0% (7/174)	3.6% (4/110)	4.7% (3/64)
Drug use	4.1% (11/269)	2.9% (5/174)	2.7% (3/110)	3.1% (2/64)
Any chronic illness	36.1% (97/269)	32.2% (56/174)	32.7% (36/110)	31.3% (20/64)
PIH/hypertension	15.2% (41/269)	14.5% (26/174)	14.6% (16/110)	15.6% (10/60)
Maternal infection	5.6% (15/269)	5.8% (10/174)	7.3% (8/110)	3/1% (2/64)
Breech presentation	21.6% (58/268)	21.8% (38/174)	20.0% (22/110)	25.0% (16/64)
Placental pathology	11.2% (30/269)	13.8 (24/174)	13.6% (15/110)	14.1% (9/64)
Preeclampsia	24.9% (67/269)	12.1% (21/174)***	13.6% (15/110)*	9.4% (6/64)**
PPROM	40.2% (107/266)	42.9% (73/170)	43.5% (47/108)	41.9% (26/62)
Antenatal steroids	84.9% (225/265)	82.6% (142/172)	82.6% (90/109)	82.5% (52/63)
Antenatal antibiotics	43.8% (113/258)	54.2% (90/166)*	55.7% (59/106)*	51.7% (31/60)
Multiple gestations	28.6% (77/269)	23.0% (40/174)	23.6% (26/110)	21.9% (14/64)
Abnormal FHR	13.8% (37/269)	9.8% (17/174)	9.1% (10/110)	10.9% (7/64)
Cesarean section	68.6% (181/264)	67.1 (114/170)	70.4% (76/108)	61.3% (38/62)
Apgar at 1 min <3	24.9% (67/269)	12.1% (21/174)***	13.6% (15/110)*	12.7% (8/63)*
Apgar at 5 min <5	3.4% (9/267)	8.1% (14/173)*	5.5% (6/110)	12.7% (8/63)**
SGA	9.3% (25/268)	9.3% (16/173)	10.1% (11/109)	7.8% (5/64)
Intubation at birth	29.8% (79/265)	66.3% (114/172)***	61.1% (66/108)***	75.0% (48/64)***
Surfactant use	34.5% (91/264)	66.5% (115.173)***	61.5% (67/109)***	75.0% (48/64)***
Dexamethasone	5.7% (15/264)	28.2% (48/170)***	22.4% (24/107)***	38.1% (24/63)***
MV	31.2% (84/269)	77.0% (134/174)***	69.1% (76/110)***	90.6% (58/64)***
CPAP	87.4% (235/269)	93.1% (162/174)	91.8% (101/110)	95.3% (61/64)*
PDA	21.6% (58/268)	52.9% (92/174)***	46.4% (51/110)***	64.1% (41/64)***
Sepsis	16.0% (43/269)	32.8% (57/174)***	30.0% (33/110)**	37.5% (24/64)***
Thrombocytopenia	5.2% (14/268)	19.5% (34/174)***	17.2% (19/110)***	12.4% (15/64)***
NEC	1.1% (3/269)	9.8% (17/174)***	10.0% (11/110)***	9.4% (6/64)***
Congenital anomalies	8.9% (24/269)	19.0% (33/174)**	18.2% (20/110)*	20.3% (13/64)**

As shown in Table 1, the demographic characteristics and most antenatal and intrapartum factors were comparable between the study groups. Infants in Group 1 had higher GA and BW than those in Group 2, Group 2A, and Group 2B. The rate of preeclampsia was higher in Group 1 than in Group 2, Group 2A, and Group 2B. A higher proportion of mothers in Group 2 and Group 2A received intrapartum antibiotics than those in Group 1. A higher proportion of infants in Group 1 were born with a one-minute Apgar score <3 than those in Group 2, Group 2A, and Group 2B. However, an Apgar score <5 at five minutes, intubation at birth, use of surfactant, dexamethasone, and mechanical ventilation were more prevalent in Group 2, Group 2A, and Group 2B than in Group 1. Additionally, more infants in Group 2, Group 2A, and Group 2B were diagnosed with PDA, sepsis, mild congenital anomalies, thrombocytopenia, and NEC than in Group 1.

Factors predicting survival without any disease (Group 1), with one (Group 2A), and more than one (Group 2B) preterm birth-related morbidities

Among the 33 factors in the univariate analysis (Table 1), 15 were included in each model (Table 2).

**Table 2:** The models depicted significant factors for predicting neonatal survival without, with one, or more than one of the studied conditions GA: Gestational age; BW: Birth weight; DART: Dexamethasone: A Randomized Trial; AB: Antibiotics; MV: Mechanical Ventilation; PDA: Patent Ductus Arteriosus; NEC: Necrotizing Enterocolitis. Factors and area under the curve (AUC) are presented as Odds Ratio and 95% Confidence Intervals. Blank cells indicate non-significant values.

Factors (Yes (1) vs. No (0))	Model 1 (G1 (0) vs. G2 (1))	Model 2 (G1 (0) vs. G2A (1))	Model 3 (G1 (0) vs. G2B (1))
GA 22-27	0.68 (0.48, 0.97)		1.96 (1.21, 3.18)
BW <1,000 g			
Pre-eclampsia	1.44 (1.05, 1.97)		
Intrapartum AB		1.43 (1.10, 1.87)	
Intubation at birth			
Surfactant			
Apgar at 1 min <3			
Apgar at 5 min <5			
Congenital anomalies			
DART Protocol			
MV	0.48 (0.35, 0.98)	1.68 (1.21, 2.34)	2.99 (1.75, 5.04)
PDA			
Thrombocytopenia	0.52 (0.35, 0.79)	1.74 (1.11, 2.75)	3.22 (1.80, 5.73)
Sepsis	0.74 (0.56, 0.97)		
NEC	0.28 (0.22, 0.91)	2.1 (1.01, 4.40)	
AUC	0.82 (0.78, 0.84)	0.70 (0.61, 0.73)	0.90 (0.88, 0.93)

Data from Model 1 showed a reduced likelihood of survival free from IVH, BPD, and ROP if infants who were born at GA 22-27 weeks required mechanical ventilation and were diagnosed with thrombocytopenia, sepsis, or NEC. However, the chance for survival without any of the studied morbidities increased if the mothers of the infants were diagnosed with pre-eclampsia. An AUC of 0.82 was considered very good for distinguishing infants without a diagnosis of IVH, BPD, and ROP from those diagnosed with at least one of these morbidities.

Model 2 demonstrated that infants exposed to intrapartum antibiotics, mechanical ventilation, thrombocytopenia, and NEC were more likely to be diagnosed with one morbidity as compared to infants without any of the studied morbidities. An AUC of 0.70 indicates a good differentiation between cases with one morbidity and those without any morbidities by identifying significant predictors. Furthermore, Model 3 highlighted that birth at GA 22-27 weeks, mechanical ventilation, and diagnosis of thrombocytopenia were the primary predictors for developing more than one disease. An AUC of 0.90 indicated the model's ability to distinguish between infants at risk for survival to discharge with multiple morbidities and those discharged without BPD, IVH, and ROP. 

## Discussion

In line with our research, more than 60% of infants born before 33 weeks have survived without being diagnosed with IVH, BPD, or ROP, which aligns with other reports showing survival rates without various significant preterm birth-related morbidities in infants born between 24 and 30 weeks [5], 22 and 32 weeks [6], and 23 and 32 weeks [7]. As in those reports, we too have highlighted the primary role of GA in predicting preterm infant survival without significant morbidities, but did not find an independent effect of gender [6], Apgar scores [5], intubation [5], SGA weight, and multiple pregnancies [5].

The regression model revealed an increased likelihood for survival without IVH, BPD, and ROP in infants exposed to maternal pre-eclampsia, a factor not previously studied while defining disease-free survival of preterm infants. Several studies have examined the role of pre-eclampsia in the development of IVH, BPD, and ROP as a single condition. These studies have reported inconsistent findings, such as a decreased risk for IVH [20], BPD [21], and ROP [22]; an increased risk for BPD [23] and ROP [24]; or no effect on the development of BPD [25]. A study [7] aimed at developing a resiliency model for survival of neonates with a GA of less than 32 weeks found that 24.7% of surviving infants without significant morbidities were born to mothers with pre-eclampsia. In contrast, only 10.5% of infants who did not survive were born to mothers with pre-eclampsia. This highlights the importance of further investigation into how pre-eclampsia affects preterm outcomes.

Additionally, our study identified an increased chance of survival without any IVH, BPD, or ROP if the infants had not been placed on mechanical ventilation and were not diagnosed with sepsis, thrombocytopenia, or NEC. Several studies have analyzed the role of these factors in the development of IVH, BPD, and ROP, but not for survival free of all these morbidities in infants born prematurely. It has been shown that the duration of mechanical ventilation increased the risk for ROP development and the prediction of the need for ROP treatment [26]. Animal studies identified mechanical ventilation-associated increased risk of cerebral inflammation and white matter injury in the immature brain [27]. Furthermore, any abnormal platelet levels were identified as a single marker for the development of BPD [28], ROP [29], and IVH [30]. Our previous study [31] found a clear link between the development of IVH and lower GA and thrombocytopenia, regardless of the severity of thrombocytopenia or the postnatal age at which it begins. Culture-proven sepsis, especially when recurrent, was identified as a factor that increased the risk for IVH, BPD, and ROP [23,32,33]. There is also a possible link between NEC and IVH-related neurodevelopmental outcomes [34], ROP [35], and the contribution of the NEC-related gut-lung axis to the pathophysiology of BPD [36].

In this study, we also examined factors predicting the number of morbidities diagnosed in surviving preterm neonates. The diagnosis of a single morbidity was predicted by exposure to intrapartum antibiotics, mechanical ventilation, and the diagnosis of thrombocytopenia or NEC. The relationship between the administration of intrapartum antibiotics and the development of one of the studied morbidities is not yet fully understood. The use of antibacterial therapy was associated with a diagnosis of clinical chorioamnionitis, which was associated with an increased risk for BPD [37] and IVH [38], but meta-analysis has shown that other factors may influence the identified associations. Consequently, the causal relationship between the use of intrapartum antibiotics and the development of BPD and IVH is not definitive. As mentioned earlier, mechanical ventilation, thrombocytopenia, and NEC are significant factors associated with an increased risk for BPD [28,36], IVH [34], and ROP [26,29,35]. Based on the AUC, factors that discern less than 80% of infants with one morbidity from those without any studied disease have limited clinical utility [39]. Mechanical ventilation and thrombocytopenia emerged as powerful predictors for diagnosing multiple morbidities, demonstrating an even more substantial impact than that seen in Model 2, which examined factors related to the development of at least one studied morbidity. In addition, the possibility of the diagnosis of more than one morbidity increased on average by two times for infants born with GA of 22-27 weeks compared to those born with GA of 28-32 weeks. An AUC of 0.90 indicated an excellent level of discrimination for surviving infants diagnosed with more than one morbidity compared to those who survived to discharge free of IVH, BPD, and ROP by extremely preterm gestation, mechanical ventilation, and thrombocytopenia.

Our study findings emphasize the importance of considering multiple factors for further exploration when analyzing the likelihood of diagnosing morbidity related to preterm birth in infants who survive to discharge. In addition to the GA and BW, a better understanding of other independent factors that forecast major preterm birth-related morbidities could be important not only for clinical management but also for parental counseling. Additionally, the study findings open new possibilities for understanding and predicting survival outcomes in preterm infants and provide valuable insights for further research and potential interventions.

Study limitations

The first limitation of the study was its retrospective design, which may introduce selection bias. To mitigate this risk, we selected the study sample from patients admitted to the NICU. We applied strict selection criteria independent of other factors, including GA and survival status. Additionally, we standardized data collection across all participants, regardless of the outcome, to prevent misclassification bias. We also utilized EMRs. This reliable tool provided us with more accurate information, particularly for gathering maternal, intrapartum, and neonatal factors, in contrast to the International Classification of Diseases (ICD), which is known to have a variety of errors in depicting clinical data [40]. The second limitation was the insufficient sample size in Group 2A (n=110) for identifying factors predicting the type of single morbidity (IVH, BPD, or ROP). Moreover, the sample size in Model 3, fell below the critical threshold of 10 EPV, which is likely to significantly undermine the accuracy of the logistic regression coefficient. It is important to emphasize that while EPV values between five and nine may be deemed acceptable, relying on such values carries inherent risks and should not be overlooked in the analysis [41,42]. This highlights the need for further research to explore this area more comprehensively. Lastly, the findings of this study are derived from a single setting. Approximately 70% of the study population involved non-White participants, and around half were on Medicaid or uninsured, which limits the generalizability of the results to broader populations.

## Conclusions

The chance for survival to discharge without IVH, BPD, and ROP in immature infants is significantly reduced when they are born at extremely preterm gestations, require mechanical ventilation, or face serious comorbidities such as thrombocytopenia, sepsis, or NEC. Among these, extremely preterm gestation, mechanical ventilation, and thrombocytopenia are the main predictors for the diagnosis of more than one of the studied morbidities, which is of utmost importance in understanding the health outcomes of immature infants. While the defined models reveal either very good or excellent levels of discrimination for the outcomes studied, further research is essential to validate these findings for clinical use.
